# Editorial: The Interplay Between Consciousness and Attention in Atypical Contexts of Experience

**DOI:** 10.3389/fnins.2022.913913

**Published:** 2022-04-29

**Authors:** Andrea Nani, Javeria Ali Hashmi

**Affiliations:** ^1^GCS-fMRI, Department of Psychology, Koelliker Hospital, University of Turin, Turin, Italy; ^2^FOCUS Lab, Department of Psychology, University of Turin, Turin, Italy; ^3^Department of Anesthesia, Pain Management and Perioperative Medicine, Dalhousie University, Halifax, NS, Canada

**Keywords:** consciousness, attention, altered state of awareness, hypnosis, psychedelics, neurological disorder, psychiatric disorder

During the last three decades, our understanding of the brain processes that underlie consciousness and attention has significantly improved. Many studies in perception science have employed sophisticated experimental paradigms to dissociate conscious perception from attention and have shown that these mental faculties are supported by distinct (albeit strictly intertwined) brain processes (Nani et al., 2019). Observations where consciousness occurs without attention and where attentional processes can occur without conscious awareness have been examined in rigorous experimental contexts (Tsuchiya and Koch, 2008). It is well-known that percepts can be experienced in the absence of sense data during dreams, altered mental states, under the influence of psychogenic drugs, and hypnosis (Yaden et al., 2021). However, there is still little understanding about the interplay of these higher-order faculties when altered states of consciousness occur in both normal and clinical conditions such as schizophrenia, bipolar disorder and other neurological or psychiatric disorders.

The aim of this Research Topic has been to investigate the interplay between consciousness and attention in different contexts, especially with regard to abnormal and/or pathological conditions of experience. The six articles that constitute the Topic cover a broad scope of these atypical contexts, ranging from altered perceptions in normal individuals to those caused by psychiatric diseases.

Vis et al. offer a systematic review of case reports about the hallucinogen-persisting perception disorder (HPPD) in order to define its phenomenology, associated (psycho)pathology, and development. The authors point out that most symptoms of HPPD are non-visual and/or characteristic of Alice in Wonderland syndrome, which is in contrast with the DSM-5 diagnostic criteria for this disorder. HPPD appears to be mainly characterized by changes in the content of consciousness and an attentional shift from exogenous to endogenous phenomena. The authors offer suggestion on how the DSM-5 diagnostic criteria could be revised.

Bachmann puts forward an opinion article about attention and “normal” hallucinations, that is, hallucinations occurring in contexts where individuals being in a normal state of mind experience nonetheless objects that are not actually present. Discussing the literature about this topic Bachmann clarifies the relationship between attention and hallucinatory experiences. Of note, hallucinated experiences in normal individuals can be associated with iconic memory post-cueing, spatial-attentional pre-cueing, and spatially divided attention without pre-cueing. Finally, the author discusses a taxonomy of types of contents in “normal” hallucinations.

Hare proposes an interesting model operating at the level of functional networks of how specific sensory representations can enter conscious awareness in schizophrenic patients. It is not well-understood yet how brain activity can give rise to aberrant conscious perceptions; in particular, there is no theory of hallucinations that identifies a “selection mechanism” for their conscious processing. Within the framework of the global workspace and the psychopathological context of schizophrenia, the author suggests a selection mechanism based on the salience network and an abnormal selection of sensory representation in schizophrenic patients, which are directly broadcast into the global workspace instead of being previously filtered.

Munoz Musat et al. discuss an electroencephalography study about hypnotic induction of deafness to elementary sounds. The atypical contexts of experience brought about by hypnosis are a unique opportunity to investigate how top-down effects can influence conscious and non-conscious processes. The authors propose that hypnotic suggestion can cause a disconnection between auditory areas and other cortical areas. This interpretation is then discussed in relation to three possible scenarios: (a) that early processing of auditory information may be preserved and unaffected by hypnotic suggestion, (b) that an inhibitory process mediated by the anterior cingulate cortex might prevent conscious access to sounds, and (c) that modular and unconscious representations of sounds may be functionally disconnected within the global neuronal workspace.

Cortese et al. discuss several cases of unresponsive wakefulness syndrome in which the nociception coma scale was used to evaluate the level of consciousness and its evolution. The assessments were performed in rigorous and reproducible contexts: at the same time of the day and in a room with constant levels of humidity, light and temperature, and absence of transient noise. After this treatment, a significant number of patients showed improvements in their level of consciousness rates. Therefore, the authors conclude that a careful evaluation of responses to nociceptive stimuli in patients with disorders of consciousness could constitute an effective procedure in assessing the likelihood of the transition from vegetative state to minimal conscious state.

Stoliker et al. discuss how psychedelics can modulate the development of belief updating and enable access to different beliefs as to how we make sense of reality. The authors hypothesize that this process may alter consciousness and favor the condition known as “ego dissolution.” According to the authors, the alteration of consciousness and attention produced by psychedelics have a common mechanism of reduced precision of Bayesian belief updating, which may prove to be a promising method of investigating both consciousness and attention.

Articles in this Research Topic collection contributed insightful ideas and discussions to the understanding of the interplay of consciousness and attention in atypical context of experience, an intriguing field that undoubtedly deserves to be more explored by future research.

## Author Contributions

AN drafted and wrote the editorial. JH revised it. All authors contributed to the article and approved the submitted version.

## Conflict of Interest

The authors declare that the research was conducted in the absence of any commercial or financial relationships that could be construed as a potential conflict of interest.

## Publisher's Note

All claims expressed in this article are solely those of the authors and do not necessarily represent those of their affiliated organizations, or those of the publisher, the editors and the reviewers. Any product that may be evaluated in this article, or claim that may be made by its manufacturer, is not guaranteed or endorsed by the publisher.

## References

[B1] Nani A. Manuello J. Mancuso L. Liloia D. Costa T. Cauda F. (2019). The neural correlates of consciousness and attention: two sister processes of the brain. Front. Neurosci. 13. 10.3389/fnins.2019.01169 31749675 PMC6842945

[B2] Tsuchiya N. Koch C. (2008). “The relationship between consciousness and attention,” in The Neurology of Consciousness: Cognitive Neuroscience and Neuropathology, eds G. Tononi, and S. Laureys (Oxford: Elsevier Academic), 63–78. 10.1016/B978-0-12-374168-4.00006-X

[B3] Yaden D. B. Johnson M. W. Griffiths R. R. Doss M. K. Garcia-Romeu A. Nayak S.. (2021). Psychedelics and consciousness: distinctions, demarcations, and opportunities. Int. J. Neuropsychopharmacol. 24, 615–623. 10.1093/ijnp/pyab026 33987652 PMC8378075

